# Delayed onset interstitial nephritis following multiple wasp stings

**DOI:** 10.4103/0971-4065.53326

**Published:** 2009-04

**Authors:** J. B. Ghosh, M. Roy, A. K. Bala

**Affiliations:** Department of Pediatric Medicine, Institute of Post-graduate Medical Education and Research, Kolkata, India; 1 Department of Pediatric Medicine, North Bengal Medical College and Hospital, Darjeeling, India

**Keywords:** Interstitial nephritis, wasp sting, acute renal failure

## Abstract

A wasp sting rarely causes delayed / late onset hypersensitivity reaction. Although unknown, the mechanism of such a delayed hypersensitivity reaction is thought to be an immunologically mediated, type III hypersensitivity reaction with the deposition of immune complexes and activation of the complement system. We present here the case of a seven year-old girl with acute interstitial nephritis following multiple wasp stings. To the best of our knowledge, this is the first such report of delayed presentation in a child from India.

## Introduction

Two different types of hypersensitivity reactions are possible after a wasp sting: commonly, (1) IgE-mediated immediate type I hypersensitive reaction (anaphylaxis) and rarely, (2) Delayed type III hypersensitive reaction with the deposition of immune complexes and activation of the complement system. We report here the case of a seven year-old girl with delayed onset acute renal failure (ARF) following multiple wasp stings. Acute interstitial nephritis was diagnosed by renal biopsy and she was treated with supportive measures.

## Case Report

A seven year-old girl presented with swelling of her body and decreased urine output since the last two days. History revealed that five days before the onset of the disease, the patient had been stung by a swarm of wasps. Immediately after the stings, there were only local symptoms without any systemic features. She was taken to a nearby hospital and treated with chlorpheniramine maleate, ranitidine, and paracetamol. Except for pain and redness at the sting sites, she remained asymptomatic; however, swelling and decreased urine output started at the end of the 5^th^ day. There was no family history of atopy or asthma or any previous incidence of insect sting(s).

On admission, the patient was found to be irritable and edematous. She had 20–25 partially healed scar marks mostly over the face, throat, and scalp. Her pulse was 120/min with an average volume, blood pressure of 110/74 mm Hg, 98% oxygen saturation, and temperature of 38.5°C. There was no evidence of anemia, jaundice, red or black colored urine, or bleeding manifestations. Her systemic examinations did not yield any remarkable findings.

An initial laboratory workup revealed: hemoglobin 11.5 g/dL, total leukocyte count 15,500 (neutrophil: 56%, lymphocyte: 11%, monocyte: 01%, eosinophil: 32%), platelet count 2,10,000/cu. mm. The erythrocyte sedimentation rate was 52/mm in the 1^st^ hour, reticulocyte count of 2%. Routine urine examination showed 50–55 RBCs/high power field (HPF), 10–15 WBCs/HPF with significant numbers of eosinophils, and albumin was 3 plus; urine and blood culture were sterile. The blood urea level was 96 mg/dL, creatinine 3.5 mg/dL, serum Na 128 mEq/L, and K 5.5 mEq/L. Liver function test results were within normal limits. The serum creatine phosphokinase level was 60 U/L (normal: < 17–167 U/L) and the LDH level was 300 U/L (normal: 240–420 U/L). Serum haptoglobin and plasma and urine hemoglobin levels were within normal limits. Urine myoglobin was absent (normal: 0–7 ng/mL).

An electrocardiogram and chest radiographs showed no abnormalities. Ultrasonography of the abdomen revealed normal-sized kidneys with normal echotexture and preserved corticomedullary differentiation; no other abnormality was detected. A kidney biopsy revealed a mild interstitial infiltration with eosinophilic and a few plasma cells.

A diagnosis was made of ARF due to delayed hypersensitive reaction following multiple wasp stings. The patient was treated with antihistaminic (both H1 and H2 blockers) agents and prednisolone. The patient's urine output started improving on the 3^rd^ day after admission, but the serum creatinine peaked to a level of 10.5 mg/dL on the 11^th^ day. She received several sessions of peritoneal dialysis until the 14^th^ day; the serum creatinine level gradually came down over the next seven days.

Follow-up after four weeks revealed the persistence of deep punched out areas at the sites of the stings. The patient's blood pressure, urine analysis, and serum biochemistry were all normal.

## Discussion

The medically important groups of hymenoptera are the Apoidea (bees), Vespoidea (wasps, hornets, and yellow jackets), and Formicidae (ants).

As in the case of other insect stings, anaphylactic reactions in the case of wasp stings are due to IgE-mediated, type 1 hypersensitivity reactions which may occur even after a single sting. A mild anaphylactic reaction consists of nausea, abdominal cramping, generalized urticaria, flushing, and angioedema. Serious reactions characterized by upper airway edema, bronchospasm, hypotension, and shock, may be rapidly fatal but are rare.[2]

Severe reactions after hymenoptera stings often begin within ten min of the sting(s) and only rarely develop after five hours.[2] And very rarely, delayed systemic reactions such as serum sickness, arthus reaction, vacuities, neuritis, nephrotic syndrome, hepatic failure, acute renal failure, myocardial infarction or cardiac arrhythmias, thrombocytopenic purpura, and severe neurological disturbances have been reported.[1–4]

The mechanisms of those delayed reactions have not been fully understood but are thought to be nonIgE-mediated immunological reactions (type III hypersensitivity reaction) with the deposition of immune complexes and activation of the complement system.

The toxic principles of hymenoptera include active amines like histamine, melittin, apamine, phospholipases A_1_, hyaluronidase, acid phosphatase, and the degranulating peptide, mastoparan. These components have direct and indirect cytotoxic (hepatic, renal, and myocyte membrane), hemolytic, neurotoxic, and vasoactive properties, which can cause intravascular hemolysis and rhabdomyolysis.[5,6] Phospholipase A_2_ is believed to trigger the release of arachidonic acid from lipid in the cell membrane, which initiates the production of inflammatory eicosanoids. Hyaluronidase in the venom causes breakdown of chondroitins and hyaluronic acid in the connective tissues, facilitating the spread of the venom.

Wasp venom can cause ARF by several mechanisms, which include acute tubular necrosis, acute interstitial nephritis, and pigment nephropathy resulting from rhabdomyolysis (myoglobinuria) or intravascular hemolysis (hemoglobinuria) and hypotension caused by anaphylactic reaction.[7,8] In our patient, there was no evidence of intravascular hemolysis, rhabdomyolysis, or hypotension. Except for mild local symptoms, the patient was quite well for a few days after being stung. Her laboratory investigations revealed blood eosinophilia with eosinopiliuria and features of interstitial nephritis on kidney biopsy. Zhang *et al*. reported that acute tubulointerstitial nephritis could lead to ARF following a wasp sting.[9] Shrma *et al.* have also reported a similar case.[10] Similarly, our patient was absolutely all right before being stung and was not taking any nephrotoxic drug chronically. Also, she was totally improved by kidney supportive measures along with oral prednisolone therapy, so her AIN was probably caused by a delayed hypersensitive reaction to the wasp venom. On the other hand, Sakhuja *et al.* had postulated that direct toxic injury could be one of the possible mechanisms of ARF following wasp envenomation.[11]

As seen in our patient, the management of systemic manifestations of wasp sting is usually supportive because of the nonavailability of any specific antivenin.

## Conclusion

The present case highlights that physicians should be aware of the entity of delayed onset hypersensitive reaction following hymenoptera stings.
